# TLR4 signaling in the development of colitis-associated cancer and its possible interplay with microRNA-155

**DOI:** 10.1186/s12964-021-00771-6

**Published:** 2021-09-03

**Authors:** Jie Guo, Mengfan Liao, Jun Wang

**Affiliations:** 1 Hubei Province Key Laboratory of Occupational Hazard Identification and Control, Wuhan University of Science and Technology, Wuhan, China; 2 New Medicine Innovation and Development Institute, Department of Pharmacy, College of Medicine, Wuhan University of Science and Technology, Wuhan, China

**Keywords:** Toll like receptor 4, MicroRNA-155, Inflammatory bowel disease, Colitis-associated cancer, Positive feedback loop

## Abstract

Ulcerative colitis (UC) has closely been associated with an increased risk of colorectal cancer. However, the exact mechanisms underlying colitis-associated cancer (CAC) development remain unclear. As a classic pattern-recognition receptor, Toll like receptor (TLR)4 is a canonical receptor for lipopolysaccharide of Gram-negative bacteria (including two CAC-associated pathogens *Fusobacterium nucleatum* and *Salmonella*), and functions as a key bridge molecule linking oncogenic infection to colonic inflammatory and malignant processes. Accumulating studies verified the overexpression of TLR4 in colitis and CAC, and the over-expressed TLR4 might promote colitis-associated tumorigenesis via facilitating cell proliferation, protecting malignant cells against apoptosis, accelerating invasion and metastasis, as well as contributing to the creation of tumor-favouring cellular microenvironment. In recent years, considerable attention has been focused on the regulation of TLR4 signaling in the context of colitis-associated tumorigenesis. MicroRNA (miR)-155 and TLR4 exhibited a similar dynamic expression change during CAC development and shared similar CAC-promoting properties. The available data demonstrated an interplay between TLR4 and miR-155 in the context of different disorders or cell lines. miR-155 could augment TLR4 signaling through targeting negative regulators SOCS1 and SHIP1; and TLR4 activation would induce miR-155 expression via transcriptional and post-transcriptional mechanisms. This possible TLR4-miR-155 positive feedback loop might result in the synergistic accelerating effect of TLR4 and miR-155 on CAC development.

**Video abstract**

**Supplementary Information:**

The online version contains supplementary material available at 10.1186/s12964-021-00771-6.

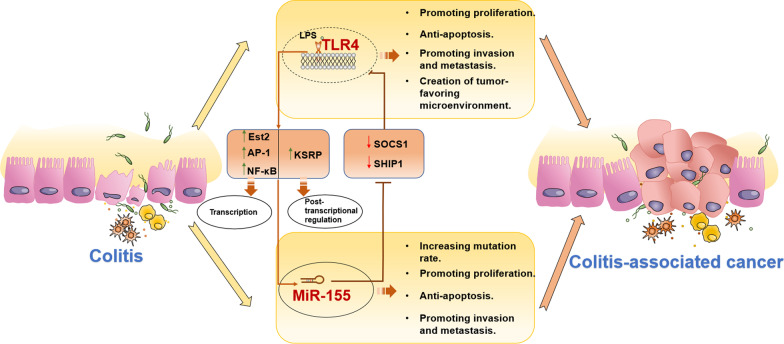

## Background

The vital roles of persistent infection and chronic inflammation in driving the initiation and progression of multiple malignancies, including cervical cancer, prostate cancer, liver cancer, gastrointestinal cancer, etc. have been acquired [1–6]. Persistent stimulation of epithelial cells and infiltration of immune cells caused by oncogenic pathogen infection and uncontrollable inflammation may create a tumor-favouring cellular microenvironment [5, 7]. Currently, colorectal cancer (CRC) is considered as the 3rd most common malignancy and the 4th leading cause of cancer-related deaths worldwide [8]. Despite that the exact etiology of CRC is still unknown, accumulative evidences have supported that patients with inflammatory bowel diseases (IBD), a group of chronic gut inflammatory disorders including ulcerative colitis (UC) and Crohn’s disease (CD), are at a clearly increased risk for the CRC development [9–11]. IBD has been believed as an independent risk factor of CRC, the incidence of CRC in IBD patients has been reported to be up to 60% higher than that in the general population [12]. In particular, population-based evaluations suggested that the risk of CRC in patients with UC was between two- and threefold that of the general population [11]. Approximately 8% of UC patients developed CRC in 20 years and 18% in 30 years of disease [13]. Moreover, the local alteration of intestinal microbiota composition and metabolic activity has been linked to chronic inflammation in IBD and cancer development in colitis-associated cancer (CAC) [11]. It has been demonstrated that some specific species such as *Fusobacterium nucleatum* (a Gram-negative anaerobic bacterium) and *Salmonella* (a Gram-negative facultative anaerobic bacterium) might be the potential pathogens that play key roles in the progression of CAC [4, 11, 14]. Nevertheless, the molecular mechanisms of CAC development remain unclear.

As the most important pattern-recognition receptors (PPRs), Toll-like receptors (TLRs) could recognize the pathogen-associated molecular patterns (PAMPs) carried by diverse microorganisms and the danger-associated molecular patterns (DAMPs) derived from stressed or damaged cells, and are responsible for the activation and association of innate and adaptive immune responses [15, 16]. TLRs have been identified as main components of infection diseases, innate immunity, inflammatory conditions [15, 16], and importantly, inflammation-mediated tumorigenesis [15, 17, 18]. Up to date, different members of TLR family have been demonstrated to contribute to the involvement of inflammation in cancer progression [18–22]. For example, it has been reported that the expression of TLR2 gradually increased from normal mucosa, to *Helicobacter pylori*-gastritis, to metaplasia, to dysplasia [19]. Hong et al. [20] have demonstrated the role of TLR3/7-mediated signaling in the induction of CAC using a well-established murine model of azoxymethane (AOM)/dextran sulfate sodium (DSS) treatment. The role of TLR7 and TLR8 expression and signaling in chronic pancreatitis-linked pancreatic carcinogenesis has also been determined [21, 22].

In particular, more and more evidences [7, 23, 24] have shown that the TLR4-mediated signaling might act as a pivotal pathogen-activated tumor signal pathway and an important carcinogenic mechanism involved in the development of CAC. As a key member of TLR family and a classic inflammatory mediator, TLR4 acts as a bridge molecule between innate and adaptive immunity, as well as between infection and inflammation [25]. Especially in the gut, a high density of luminal microbes and the abundant PAMPs coexist with the intestinal mucosa [26]. TLR4 has been well-accepted as the main PRR as well as the canonical receptor for lipopolysaccharide (LPS) of Gram-negative bacteria, a group of predominant gut pathogens [27, 28]. Moreover, the intestinal invasion of CAC development-related pathogens such as *F. nucleatum* and *Salmonella* has been reported to target and activate TLR4 signaling [4, 11, 29]. As a type I transmembrane glycoprotein receptor containing 839 amino acids [30], TLR4 is widely expressed on various immune cells, epithelial/endothelial cells and tumor cells, etc. [31] Several auxiliary molecules including LPS binding protein (LBP), cluster of differentiation 14 (CD14), and myeloid differentiation factor 2 (MD-2) are required in the TLR4 receptor complex as co-receptors for TLR4 [31]. Once recognized, LPS can be transferred to cell surface CD14 by LBP, then bond with the TLR4/MD-2 receptor complex [31]. Subsequently, the LPS/MD-2/TLR4 complex recruits two distinct intracellular adaptor proteins including myeloid differentiation primary response gene 88 (MyD88)/MyD88 adaptor-like (Mal) and TIR-domain-containing adapter-inducing interferon-β (TRIF)/TRIF-related adaptor molecule (TRAM), then triggers activation of two parallel signaling pathways, MyD88-dependent pathway and MyD88-independent pathway, resulting in transcription of inflammatory cytokines, such as tumor necrosis factor-α (TNF-α), interleukin-6 (IL-6), IL-1, and secretion of type I interferons [32]. It has been reported that the TLR4/MD2 expression levels on intestinal epithelial cells are relatively low under normal conditions, but significantly upregulated during the development of IBD [33]. More importantly, disturbance of TLR4 pathway has been considered as one of the unique aspects of IBD-related colorectal tumorigenesis [34]. In this review, we summarized the current findings about the potential role of TLR4 during CAC progression, and its potential association with microRNA (miR)-155, a CAC-related miRNA.

## Up-regulation of TLR4 in colitis and CAC

In healthy adult mammals, TLR4 has been found to be lowly expressed in the intestinal epithelium, thereby limiting excessive inflammatory responses directing towards the numerous microbial pathogens in the enteric cavity [33, 35]. However, the expression of TLR4 has been frequently reported to be markedly upregulated in the inflamed intestinal mucosa [36–39]. The significantly enhanced expression of TLR4 transcripts and cell surface protein was demonstrated in the crypt epithelial cells isolated from mucosal samples of UC patients when compared to normal controls [37]. Tan et al. [38] also reported that TLR4 was expressed on inflammatory cells in the intestinal lamina propria and submucosa of patients with UC at active phase, but not detected in healthy controls. Moreover, TLR4 expression levels were positively correlated with disease activity indices, endoscopy scores and histopathological scores [38]. In a study involving 41 patients with active or inactive IBD [39], TLR4 mRNA was shown to be significantly upregulated in biopsy tissues, specifically from patients with signs of active UC, the expression of which was approximately 13 times higher than healthy controls.

In addition, studies [40, 41] showed that the mRNA and protein of TLR4 were overexpressed in colonic mucosa of CRC patients compared with the controls. TLR4 expression in CRC was significantly correlated with tumor stage and cancer-related survival outcomes [42]. More importantly, Fukata et al. [23] have demonstrated that the epithelial TLR4 expression was gradually increased from the lesions of active UC to those of low-grade dysplasia, high-grade dysplasia and CAC in human samples, thus suggested a critical contribution of epithelial TLR4 in the induction of inflammation-induced intestinal tumorigenesis. This clinical finding has been supported by animal experimental data [43]. The gene expression of TLR4 was revealed to be increased in classic mouse models of acute and chronic colitis induced by DSS, and the highest level of TLR4 expression was seen in colonic tumors induced by AOM/DSS [43]. These results collectively showed that TLR4 might at least act as a biomarker for the progression of CAC.

## Role of TLR4 in CAC development and its possible mechanisms

Further gain- or loss-of-function experiments [23, 43–45] demonstrated the gradually upregulated TLR4 during CAC development might not only act as a biomarker, but also play an important role in promoting colitis-associated tumorigenesis. Fukata et al. [23] found that, after administration of AOM/DSS, the number of colonic tumors in villin-TLR4 mice, a transgenic mouse model carrying a constitutively active TLR4 in the intestinal epithelium, was significantly higher than that in wild type (WT) mice. Using an antagonist TLR4 antibody, the study team [23] demonstrated that the inhibition of TLR4 markedly suppressed the development of colonic tumors in the WT mice. Whereas, in the TLR4^−/−^ mice treated with AOM/DSS, the incidence of colorectal neoplasia as well as the size and severity of dysplasia were found to be significantly decreased compared to those in WT mice [43]. In addition, Makkar et al. [44] induced syngeneic tumor isografts by injection of mouse colon cancer CT26 cells into BALB/c mice, and found that TLR4^−/−^ CT26 cell tumors grew more slowly than WT CT26 tumors. Shi et al. [45] reported that deficiency of TLR4 significantly reduced the number of intestinal tumors in Apc^Min/+^ mice, a mouse model of spontaneous intestinal tumorigenesis.

The possible mechanisms underlying the potential carcinogenic role of TLR4 in CAC initiation and development might be as follow.

### Proliferation-promoting effect of TLR4

In a DSS-induced acute experimental colitis model, TLR4 knockout mice were found to be deficient in the ability of epithelial repair in response to DSS-induced injury, suggesting that the proliferation-promoting effect of TLR4 signaling was required for basal resistance against DSS-induced acute intestinal injury [46]. However, based on this proliferation-promoting activity, it could be speculated that long-term and excessive upregulation of TLR4 signaling might lead to the initiation and development of CAC.

In villin-TLR4 mice, overexpression of TLR4 in intestinal epithelium has been demonstrated to result in the increased epithelial proliferation, the expansion of crypt epithelial cells and the development of spontaneous duodenal dysplasia [23]. Furthermore, treating villin-TLR4 mice with AOM led to robust colonic tumorigenesis, accompanied by the dramatic proliferation of cells in tumors and surrounding tissues [47]. In human colon cancer cell lines HT-29 and SW480, CCK8 assay showed that the cell proliferation was suppressed by the inhibition of TLR4 using TLR4 siRNA [48]. Similarly, Kuo et al. [49] challenged HT-29 cells with a nonapoptotic dose of LPS, and found that the increase of cell proportion in S and G_2_-M phases following LPS challenge was eliminated by gene silencing of TLR4. In Apc^Min/+^ mice treated with *F. nucleatum*, the expression of cell cycle regulatory gene Cyclin D1 was found to be significantly decreased by inhibition of TLR4 using TAK-242 [50]. Makkar et al. [44] used BrdU as a proliferative marker, and showed that the proliferation in TLR4^−/−^ CT26 cell tumor isografts was significantly impaired compared to that in CT26 WT tumor isografts. Another study [45] also found that the number of Ki67-positive proliferative cells was much higher in tissues from the Apc^Min/+^ WT mice than in tissues from the Apc^Min/+^ TLR4^−/−^ mice. Kuo et al. [49] reported that the tumor cell proliferation in AOM/DSS-induced CAC was significantly decreased by the administration of eritoran, a TLR4 inhibitor. Furthermore, the same study [49] explored the effect of TLR4 inhibition by eritoran or TLR4 siRNA in primary cultures of colonic tumor spheroids on tumor cell proliferation in vitro, and found that TLR4 inhibition suppressed the increase of spheroid growth and the acceleration of cell-cycle progression induced by LPS. These above findings suggested that TLR4 could promote the proliferation of intestinal epithelial cells and malignant cells, thus might act as a potent proliferative driver during the development of CAC.

### Anti-apoptotic effect of TLR4

Fukata et al. [51] have found that the TLR4 knockout mice showed enhanced intestinal epithelial cell apoptosis following DSS-induced acute colitis. This anti-apoptotic effect of TLR4 has been considered to be beneficial in reducing acute intestinal injury and promoting mucosal repair [51, 52]. However, given its anti-apoptotic activity, overexpression of TLR4 during colonic inflammation could be speculated to protect malignant cells against apoptosis and further promote tumor cell growth.

In human colon cancer cell lines HT-29, SW480 and Lovo, flow cytometry analysis showed that a significantly high level of apoptosis induced by TNF-related apoptosis-inducing ligand (TRAIL) was attenuated by the typical TLR4 activator LPS [53]. Chung et al. [54] induced apoptosis of human colon cancer HCT-116, HT-29 and HCT-8 cells by oxaliplatin and 5-fluorouracil (5-FU). After treatment with oxaliplatin and 5-FU, cells were incubated with or without LPS. The results showed that the LPS treatment for 8 h significantly inhibited the apoptosis of drug-treated colon cancer cells, as evidenced by the increased expression of anti-apoptosis-related B-cell lymphoma 2(Bcl-2) family proteins and the decreased activity of pro-apoptosis caspase-3/7 [54]. In CT26 tumor isografts, the numbers of apoptotic cells and the activity of caspase-3, which were detected using TUNEL assay and immunofluorescence, respectively, were found significantly increased by TLR4 knockout [44]. In an in-vivo study conducted by Kuo et al. [55], TLR4-mut mice (TLR4-deficient mice harboring a point mutation in the TLR4 at Pro712) and their WT counterparts were used for AOM/DSS administration. Then the higher numbers of apoptotic cells per area of tumor were found in TLR4-mut mice than in WT mice. This study [55] also explored the effect of TLR4 deficiency on cell apoptosis in colonic tissues by an ex-vivo experiment. The colonic tissues of TLR4-mut or WT mice were stimulated with LPS from the mucosal side. Enhanced epithelial apoptosis was found in colonic tissues of TLR4-mut, but not WT mice. These data confirmed that TLR4 could protect colonic cancer cells from cell apoptosis in vitro and in vivo.

However, a conflicting study conducted by Li et al. [56] showed that the constitutive activation of TLR4 in intestinal epithelium resulted in an elevation of intestinal tumor cell apoptosis levels and a reduction of intestinal tumor burden in the Apc^Min/+^ mice, indicating that persistent epithelial activation of TLR4 might play a role in inhibiting intestine tumorigenesis by enhancing apoptotic signals. This finding appeared to be in contradiction with the study conducted by Kuo et al. [55] suggesting deficiency of TLR4 increased epithelial apoptosis in AOM/DSS mouse model. This contradiction might be due to the different animal models used in these studies. Li et al. [56] introduced the CD4-TLR4 transgene linked to an intestinal epithelial cell-specific promoter into APC^Min/+^ mice. The authors attributed the pro-apoptotic effect of epithelial TLR4 activation on intestinal tumor cells to the downregulation of cyclooxygenase 2 (Cox-2), a major mediator of tumor survival and growth which can impart resistance to apoptosis, in CD4-TLR4-expressing intestinal tumors [56]. However, Kuo et al. [55] observed the potential anti-apoptotic effect of TLR4 using TLR4-mutant mice harboring a spontaneous spontaneous point mutation of the TLR4 gene at Pro712His (C.C3-*Tlr4*^*LPS−d*^/J strain). The expression change of Cox-2 was not included in this study [55]. Nevertheless, Fukata et al. [51] reported that, using an AOM/DSS-induced CAC mouse model, Cox-2 expression was significantly decreased in the mucosa of TLR4-deficient mice compared to that of WT mice. The enhanced Cox-2 levels were also observed in the villin-TLR4 transgenic mice generated using plasmids containing the mouse villin promoter (pBS-Villin) and the mCD4-hTLR4 fusion gene [23], in which CD4-TLR4 construct was expressed under a different epithelial promoter than the study conducted by Li et al. [56] Therefore, further researches on the regulatory mechanisms of TLR4 on Cox-2 under different contexts (e.g. the presence or absence of inflammatory stimulation) or under different genetic backgrounds might be required to identify the role of TLR4 in regulating apoptosis of intestinal tumor cells.

### Role of TLR4 in invasion and metastasis

High expression of TLR4-mediated signaling was found to be significantly associated with a high risk of liver metastasis and poor prognosis in CRC patients [57]. Ying et al. [58] explored the role of TLR4 signaling in the metastasis potential of mouse C26 and human colon cancer HCT-116 cells, and found that silencing of TLR4 expression significantly suppressed the LPS-induced migration and invasion of C26 and HCT116 cells, which were detected using wound healing assay and transwell assay, respectively. Subsequently, the study team [58] found that stimulation of TLR4 by LPS induced down-regulation of epithelial marker E-cadherin and up-regulation of mesenchymal marker Vimentin, suggesting that activation of TLR4 signaling induced the epithelial-mesenchymal transition (EMT) phenotype in colon cancer cells. EMT-like attributes have been well-accepted to greatly contribute to invasive phenotype and metastatic capacity of the migratory subpopulation in CRC [59]. In addition, Killeen et al. [60] showed that inhibition of TLR4 in human colon cancer SW480 and SW620 cells ameliorated tumor cell invasion and adhesion to the extracellular matrix, the latter of which has been considered as a key step in the process of tumor metastasis [61]. In an in-vivo study conducted by Hsu et al. [62], LPS-treated or untreated HT-29 cells were injected into athymic nude mice. After 5 weeks of incubation, the mice injected with LPS-treated HT29 cells were found to have a significantly higher number of liver surface metastatic nodules compared with those injected with untreated HT29 cells, and this effect exerted by LPS could be attenuated by TLR4 antagonist. Similarly, using a mouse model of colon cancer cell metastasis to lungs, another study [63] showed that MD2 blockage suppressed the metastatic capacity of colon cancer cells in vivo through inhibiting TLR4/MD2 signaling. These findings collectively suggested the potential role of TLR4 in enhancing the aggressiveness and metastatic ability of CRC cells.

### Involvement of TLR4 in tumor-favouring cellular microenvironment

It has been well-accepted that the interaction between tumor cells and cells of the surrounding microenvironment could promote the progression of tumor [64]. TLR4 expression in the colonic tumor microenvironment has been found to be positively correlated with the disease progression of CRC [65]. During DSS-induced acute colitis, the production of chemokines, including C–C motif chemokine ligand 2 (CCL2), CCL20 and Chemokine C-X3-C-Motif Ligand 1 (CX3CL1), as well as the infiltration of macrophages and dendritic cells (DCs) were found to be decreased in anti-TLR4 antibody-treated mice [66]. Using bone marrow (BM) chimeric mice to construct AOM/DSS-induced CAC model, Fukata et al. [24] reported that the infiltration of neutrophils and macrophages as well as the expression of chemokines CCL2 and keratinocyte-derived chemokine were higher in WT mice engrafted with TLR4^−/−^ BM than in TLR4^−/−^ mice engrafted with WT BM, indicating that TLR4 signaling on colonic epithelial cells rather than the myeloid compartment could promote the recruitment of inflammatory cells in the tumor microenvironment of CAC. Furthermore, macrophages recruited in the tumor microenvironment could differentiate into tumor-promoting M_2_-phenotype or tumor-suppressing M_1_-phenotype [67]. Chen et al. [68] reported that inhibition of TLR4 by TAK-242 significantly suppressed the in-vitro M_2_ polarization of macrophage Raw264.7 cells induced by *F. nucleatum*. The in-vivo study in *F. nucleatum*-treated Apc^Min/+^ mice also showed that pre-treatment of TAK-242 significantly reduced the M_2_ polarization of macrophages within the tumor microenvironment [68]. These data indicated that TLR4 signaling upregulated in colitis might contribute to the creation of tumour-favouring microenvironment during the development of CAC.

## Potential role of miR-155, a CAC-related miRNA, in regulating TLR4 signaling

In recent years, based on the above potential roles of TLR4 in CAC development and progression, much attention has been focused on the regulation of TLR4 signaling in the context of colitis-associated tumorigenesis. Many TLR4-related signaling molecules, such as NF-κB, PI3K/Akt, Wnt/β-catenin, EGFR, GSK3β, Erk1/2, JNK and Nox1 [30, 38, 49, 68, 69], have been reported to be implicated in the mechanisms underlying the role of TLR4 in CAC.

In particular, as a high-profile mechanism to silence gene expression, miRNA-mediated post-transcriptional regulation has been found to contribute to the control of TLR4 signaling pathway [70, 71]. miRNAs are a group of small endogenous non-coding RNAs of ~ 22 nucleotides that regulate gene expression by binding to the 3’-untranslated regions (UTRs) of target mRNAs, leading to mRNA degradation or translation breakdown [72, 73]. Among the multiple miRNA molecules regulating TLR4 pathway, miR-155 captures our attention because of its highly similar role to TLR4 in the pathogenesis of CAC.

It has been frequently demonstrated that the miR-155 expression is markedly increased in inflamed colonic mucosa of UC patients [74–77]. The fold changes of miR-155 expression levels in inflamed human UC samples compared to the controls were reported to range from 1.22 to 2.33 [75, 76]. In DSS-induced acute colitis animal model, miR-155 antagomir dramatically distributed in colon epithelial and submucosal cells was significantly upregulated by DSS challenge, with fold change of 4.74 compared to the normal controls [78]. Importantly, El-Daly et al. [79] had applied the stepwise CAC model induced by AOM/DSS to detect the aberrant expression of miR-155 during the different stages of CAC development. The results showed that the expression of miR-155 was gradually increased as the mouse colonic tissue transformed from normal to actively inflamed to malignant state, suggesting that miR-155 might play a role in the progression of CAC. Coincidently, this dynamic change of miR-155 expression in a stepwise pattern was consistent with that of TLR4 during stages of CAC development observed by Fukata et al. [43]

As a multi-functional microRNA, miR-155 has been found to markedly promote cell proliferation and pro-inflammatory secretions, regulate the immune balance in colonic mucosa of IBD, thus contribute to the pathogenesis of experimental colitis [80, 81]. Importantly, the function of miR-155 in CAC promotion has been demonstrated in miR-155 deficient mice exposed to AOM/DSS challenge [82]. Compared to syngeneic WT mice, host miR-155 deficiency significantly decreased the tumor incidence and the multiplicity of colonic neoplasms. This study [82] also showed a similar inhibitory effect posed by miR-155 deficiency on the in-vivo growth of transplantable mouse colon cancer MC38 tumors in mice. In human colon cancer cell lines SW480 and HCT116, hypoxanthine phosphoribosyltransferase assay showed that the mutation rate was increased along with the upregulation of miR-155 expression levels during doxycycline treatment [83]. This finding established a direct link between the mutation rate and the miR-155 expression level in colon cancer cells, suggesting that miR-155 might promote colonic tumorigenesis based on its mutant phenotypes. Transfection of HCT-116 cells with miR-155 mimics significantly inhibited the cell apoptosis, and promoted the proliferation, cell cycle progression and invasive abilities [84]. Whereas, the cell growth, motility and invasion of human colon cancer cell lines SW480, DLD-1, LS174T have been found to be inhibited by knockdown of miR-155 [85]. These findings collectively suggest that miR-155 might act as an oncogenic miRNA during the development of CAC, which is also similar to the above-mentioned CAC-promoting effect of TLR4.

However, Velázquez et al. [86] presented a contrary evidence, and showed that administration of AOM/DSS to miR-155^−/−^ mice caused an increased number of polyp/adenoma and a higher grade of epithelial dysplasia than the administration to WT controls, indicating that miR-155 might protect against the development of CAC. This finding was in contradiction with the report of Chen et al. [82]. As both studies used a similar AOM-DSS administration protocol, this contradiction might be due to the difference in the experimental environment or the genetic background of experimental mice. Therefore, further research and replication should be conducted to verify the oncogenic property of miR-155 in CAC.

Of note is the potential regulatory activity of miR-155 for TLR4 signaling (Table 1). Currently, miR-155 has been considered as a critical regulator of innate/adaptive immune response and TLRs signaling [87, 88]. Marques-Rocha et al. [89] found an evidence showing that miR-155 might exert a direct inhibitory control over TLR4 expression. In this study, overexpression of miR-155 by transfection of miR-155-3p mimic significantly downregulated the expression of TLR4 in human acute monocytic leukemia THP-1 cells and THP-1-derived macrophages [89]. However, another study [90] showed that transfection with miR-155 mimic resulted in the significantly increased expression of TLR4 in human HaCaT keratinocytes. In an experimental autoimmune prostatitis model, Fu et al. [91] found that the expression of TLR4 in prostatic tissues of miR-155^−/−^ mice was lower than that of WT mice. Using miR-155^−/−^ mice and WT mice to establish permanent middle cerebral artery occlusion model, Wen et al. [92] showed that TLR4 expression was significantly decreased in the ischemic cerebral tissues of miR-155^−/−^ mice compared with that in WT mice, whereas overexpression of miR-155 induced by pAd-miR-155 transfection markedly increased the TLR4 expression in ischemic cerebral tissues. Therefore, further studies in more cell lines and more animal models should be conducted to verify the exact regulatory role of miR-155 for TLR4 signaling.

**Table 1:** The direct link between TLR4 and miR-155 in the context of different disorders or cell lines

	Cells/tissues types used in in vivo/in vitro studies	Intervention	Dose and time course of intervention	Main related outcomes	Measurement methods used to determine the main related outcomes	The studied disease conditions	References
Regulatory activity of miR-155 on TLR4 signaling	Human HaCaT keratinocytes	miR-155 mimic transfection	100 ng for 6 h	TLR4 expression was significantly increased by miR-155 overexpression	Western blot	Psoriasis	[90]
	Mouse microglia BV2 cells	miR-155 mimic transfection	1 μmol/L for 24 h	Oxygen–glucose deprivation-induced TLR4 upregulation was promoted and inhibited, respectively, by miR-155 overexpression and knockdown	Western blot	Ischemic brain injury	[92]
		miR-155 inhibitor transfection	2 μmol/L for 24 h				
	Human acute mono-cytic leukemia THP-1 cells and THP-1-derived macrophages	miR-155-3p mimic transfection	15 nM for 12 h	TLR4 was downregulated by miR-155-3p in monocytes and macrophages	qPCR	–	[89]
	Mouse brain tissues	*miR-155*−/− mice	Model animal	Marked expression of TLR4 was observed in ischemic cerebral tissue of WT mice at 24 h after middle cerebral artery occlusion, and this expression was obviously reduced in *miR-155*−/− mice	Western blot /qPCR	Ischemic brain injury	[92]
		Injection of *pAdmiR-155* into lateral cerebral ventricle of mice	Single stereotactic injection	TLR4 expression was significantly increased in ischemic cerebral tissue of miR-155-overexpressing mice compared with *pAd*-infected mice	Western blot /qPCR		
	Mouse prostate tissues	*miR-155*−/− mice	Model animal	miR-155−/− mice with prostatitis exhibited the suppressed TLR4/NF-κB pathway as compared with the WT mice with prostatitis	Western blot	Experimental autoimmune prostatitis	[91]
Inductive effect of TLR4 activation on miR-155	Mouse RAW264.7 cells	LPS treatment	100 ng/mL for 0–24 h	Among the miRNAs that were induced the most by LPS, miR-155 was on the top of the list	MicroRNA arrays	–	[93, 105, 106, 110, 123, 135, 136]
				It was validated that miR-155 and BIC/primiR-155 expression was increased in a time-dependent manner	Northern blot/qPCR		
			0–1 μg/mL for 24 h	miR-155 and BIC expression was increased in a dose-dependent manner	qPCR		
			100 ng/mL for 24 h	In cells transfected with pri-155 promoter, pri-155 promoter was drove fivefold by LPS	Luciferase assay		
			10 ng/mL for 6 h	Among the LPS-induced miRNAs, miR-155 showed the highest fold change (40-fold)	MicroRNA deep-sequencing		
	Mouse BMDMs	LPS treatment	100 ng/mL for 0–24 h	The time-dependent induction of BIC transcript and miR-155 by LPS was observed in BMDMs from WT mice	Northern blot/qPCR	–	[93, 105, 110, 123, 129, 135, 136]
			0–0.1 μg/mL for 8 h	miR-155 expression was increased in a dose-dependent manner	qPCR		
			100 ng/mL for 8 h	A strong and prolonged increase of miR-155 expression was induced by LPS in BMDMs without a concomitant increase of pri-miR155	Northern blot/Semi-quantitative RT-PCR		
		TLR4−/− BMDM	Model cell	LPS-induced miR-155 expression was decreased from approximately 40-fold activation in WT-BMDM to less than twofold in TLR4−/− BMDM	qPCR		
	Human PBMCs	LPS treatment	100 ng/mL for 0–24 h	miR-155 expression was increased eightfold by LPS in PBMC after 24 h stimulation	qPCR	–	[93, 135]
	Human monocytes	LPS treatment	100 ng/mL for 6 and 24 h	miR-155 expression was upregulated by LPS at both timepoints	MicroRNAdeep-sequencing/qPCR	–	[109]
	Mouse bone marrow-derived DCs	LPS treatment	1 ug/mL for 24 h	In response to stimulation with LPS, miR-155 expression was upregulated in mature DCs as compared with immature DCs	MicroRNA arrays/qPCR	–	[108]
	Human monocyte-derived DCs	LPS treatment	1 ug/mL for 24 h	At 24 h after stimulation, miR-155 expression was upregulated by LPS when compared to cells receiving medium alone	qPCR	–	[107]
	Human monocyte-derived Macrophage	LPS treatment	100 ng/mL for 24 h	An increase of miR-155-5p levels induced by LPS was found in monocyte- derived macrophage (fold change ~ 60)	qPCR	–	[132]
	Mouse primary microglia	LPS treatment	0.1 or 1 μg/mL for 18 h	LPS treatment at the level of 0.1 or 1 μg/mL lead to a 12-fold or 21-fold increase in the expression of miR-155, respectively	qPCR	Ischemic brain injury	[111, 113]
				The miR-155 labelling was significantly more intense in the cytoplasm of microglia cells incubated with LPS than in control cells	In situ hybridization		
	Mouse microglia N9 cells	LPS treatment	0.1, 0.5 and 1 μg/mL for 18 h and 0.1 μg/mL for different time periods (0.5, 1, 2, 4, 18 and 24 h)	miR-155 expression was induced in a dose-dependent manner, which reached a 25-fold increase in miR-155 levels for the highest LPS concentration tested	qPCR	Ischemic brain injury	[111]
				miR-155 levels continued to increase, reaching a maximum at 18 h, but showed a tendency to decrease after an incubation period of 24 h			
	Mouse microglia BV2 cells	LPS treatment	100 ng/mLfor 4 h	miR-155-3p was the most significantly upregulated miRNA (by 29-fold versus control), followed by miR-155-5p (by 9.7-fold)	MicroRNA sequencing	Brain injury	[112, 113]
			20 ng/mL for 24 h	miR-155 expression was significantly increased	qPCR		
	Mouse kupffer cells (KCs)	LPS treatment	100 ng/mL for 6 and 18 h	Alcohol-induced miR-155 expression was further augmented by in vitro LPS challenge in KCs isolated from alcohol-fed mice compared with pair-fed mice	qPCR	Alcoholic steatohepatitis	[100, 133]
	Mouse alveolar macrophages (AMs)	LPS treatment	100 ng/mL for indicated time periods (0–6 h)	miR-155 expression level was dramatically increased in response to LPS stimulation in control AMs	qPCR	Acute lung injury	[128]
	Human proximal tubule epithelial HK-2 cells	LPS treatment	5 μg/mL for 24 h	miR-155 expression was significantly elevated	qPCR	Acute kidney injury	[114, 115]
	Rat synovial fibroblast	LPS treatment	1 mg/L for 24 h	miR-155 expression was significantly higher in the LPS-treated group than in control group	qPCR	Rheumatoid arthritis	[116]
	Human umbilical vein endothelial cells	LPS treatment	0.05, 0.1 and 1 μg/L for 24 h	The expression of miR-155 was enhanced by LPS in a dose dependent manner	qPCR	–	[117]
	Mouse osteoblasts	LPS treatment	50 ng/mL for 0–48 h	miR-155 expression was upregulated in a time-dependent manner, reached a peak at 24 h	qPCR	Osteomyelitis	[118]
	Mouse pre-osteoblast MC3T3-E1 cells	LPS treatment	100 and 200 ng/mL for 0–48 h	The level of miR-155 was significantly upregulated in a time-dependent manner upon LPS stimulation compared to the control	qPCR	Osteomyelitis	[119]
	Mouse osteoclasts	LPS treatment	50 ng/mL for 24 and 48 h	The expression level of miR-155 was low in pre-OCs, whereas it was up-regulated upon LPS stimulation > 140-fold at 24 h and maintained it up to 48 h	qPCR	Inflammatory bone loss	[118]
	Human breast cancer MCF-7 cells	LPS treatment	5 ng/mL for 6 and 12 h	The level of miR-155 expression was about threefold higher at 6 h after LPS stimulation	qPCR	Breast cancer	[120]
	Human B-lymphoma BJAB cells	LPS treatment	5 μg/mL for 6 and 24 h	miR-155 levels were increased by ∼twofold after 6 h of LPS treatment and by ~ threefold at 24 h	qPCR	B-cell lymphoma	[121]
				Treatment of BJAB cells with LPS for 6 h caused a significant increase in BIC mRNA levels and BIC mRNA remained elevated after 24 h treatment	Semi-quantitative RT-PCR		
	Mouse Insulinoma MIN6 Cells	LPS treatment	5, 20, or 50 ng/mL for 6 h	miR-155-5p expression was increased	qPCR	–	[122]
	Human islet cells	LPS treatment	5 or 50 ng/mL for 6 h	miR-155-5p expression was increased	qPCR	–	[122]
	Human bronchial epithelial 16HBE cells	LPS treatment	1 μg/mL for 6 h	miR-155 expression was induced by activation of TLR4 by LPS	qPCR	Chronic airway inflammatory diseases	[124]
	Human intestinal myofibroblasts (IMF)	LPS treatment	1 μg/mL for 72 h	LPS increased miR-155 level following 72 h exposure in control-derived IMF and further upregulated miR-155 level in UC-derived IMF	qPCR	Ulcerative colitis	[131]
	Human trophoblast HTR-8/SVneo cells	LPS treatment	0–800 ng/mL for 0.5–48 h	miR-155 was increased in a time- and dose-dependent manner and the highest level of miR-155 was observed at 24 h after 100 ng/mL LPS treatment	qPCR	Pre-eclampsia	[134]
	Mouse brain tissues	Intraperitoneal injection of LPS	0.8 mg/kg single injection	miR-155 expression level was increased by LPS in comparison to the control mice	qPCR	Neuroinflammation	[125]
	Mouse tibiae	Intraperitoneal injection of LPS	5 mg/kg once a week for 3 weeks	The expression of miR-155 in the tibiae of LPS-treated mice was approximately 3.4-fold higher than that in vehicle treated ones	qPCR	Inflammatory bone loss	[118]
	Mouse myocardium tissues	Intraperitoneal injection of LPS	5 mg/kg single injection	miR-155 expression level was markedly elevated in the myocardium as early as 5 h post-LPS injection and at least persisted to 24 h	qPCR	Sepsis-induced myocardial dysfunction	[127]
	Mouse liver tissues	Intraperitoneal injection of LPS	20 mg/kg single injection	At 6 h and 12 h after LPS administration, the level of miR-155 in liver tissues showed about sixfold or sevenfold increase	qPCR	Septic liver injury	[126]
	Mouse kidney tissues	Intraperitoneal injection of LPS	20 mg/kg single injection	Renal cortex miR-155 was highly induced after LPS treatment	qPCR	Sepsis-associated kidney injury	[115]
	Mouse prostatic tissues	Injection of LPS into the prostatic urinary tract	1 mg/mL single injection	miR-155 expression levels were significantly increased in psoriasis tissues compared with normal tissues	qPCR	Chronic prostatitis	[90]
	Mouse lung tissues	Injection of LPS via the endotracheal intubation	1.25 mg/mL single injection	miR-155 expression levels in mouse lungs were significantly upregulated and reached a peak at around 6 h after LPS stimulation	qPCR	Acute lung injury	[128]
	Rat lung tissues	Injection of LPS via the endotracheal intubation	10 mg/kg single injection	miR-155 was significantly overexpressed in LPS-induced acute lung injury	qPCR	Acute lung injury	[129]

−: Not mentioned

As for the possible regulatory mechanisms, several studies [70, 92–94] have demonstrated that miR-155 could augment TLR4 signaling through targeting suppressor of cytokine signaling 1 (SOCS1), a key negative regulator of TLR4 signaling [93]. Wang et al. [94] revealed that transfection of mouse primary macrophages with miR-155 mimics enhanced TLR4 responsiveness, which was manifested as enhanced production of LPS-induced TNF-α and nitric oxide (NO); importantly, luciferase assay showed that miR-155 directly targeted 3’ UTR of *SOCS1*. Similarly, Chen et al. [93] showed that overexpression of miR-155 in VDR^−/−^ bone marrow derived macrophages (BMDMs) treated with LPS resulted in the markedly reduced expression of SOCS1, as well as a much more robust and prolonged production of pro-inflammatory cytokines TNF-α, IL-6 and IL-1β, suggesting miR-155 could enhance and prolong TLR4-mediated inflammatory responses through excessive inhibition of SOCS1. In oxygen–glucose deprivation-treated microglia BV2 cells, overexpression or knockdown of miR-155 could respectively promote or inhibit TLR4 expression, which was also accompanied by the reduced or increased expression of SOCS1, respectively [92]. These data highly suggested that miR-155 might act as a direct inhibitor of SOCS1, thus play an indirect upregluatory role for TLR4 signaling.

Interestingly, it has been reported that the expression of SOCS1 protein was significantly suppressed in peripheral blood mononuclear cells (PBMCs) of primary sclerosing cholangitis (PSC) patients compared with controls, importantly, which was accompanied by a significant enhancement of miR-155 expression indicating miRNA155-modulated SOCS1 expression [95]. In fact, PSC frequently co-occurs in patient with IBD, the presence of concomitant PSC with IBD represents a distinct disease phenotype that carries a higher risk of CRC [96, 97]. UC patients with concomitant PSC has been reported to bear a tenfold increased risk of CRC, compared to patients with UC alone [97]. Moreover, upregulation of TLR4 signal has also been found in PSC patients [98, 99]. Therefore, it is necessary to further explore the relationships among miR-155, SOCS1 and TLR4, especially in UC patients with concomitant PSC.

In addition, miR-155 has been reported to enhance TLR4 signaling by inhibiting Src homology 2 domain-containing inositol-5′-phosphatase 1 (SHIP1) [100, 101], the latter of which has been demonstrated to negatively regulate LPS/TLR4-mediated inflammatory response via suppression of LPS-induced combination between TLR4 and MyD88 [102]. MiR-155 has been demonstrated to directly bind to the 3′-UTR of *SHIP1* mRNA, and induce a significant reduction in SHIP1 expression in primary BMDMs and Raw264.7 cells [80]. Accordingly, the in-vivo study showed a significantly increased SHIP1 expression, along with decreased inflammatory responses, in the antagomiR-155-treated mice [80]. In LPS or alcohol-pretreated liver kupffer cells (KCs), Bala et al. [100] reported that overexpression and inhibition of miR-155 could increase and decrease the SHIP1 expression, respectively. Another study [101] showed that transfection of primary microglia with miR155-5p mimics significantly repressed the luciferase activity in *SHIP1* 3′ UTR, and downregulation of miR155-5p significantly attenuated TLR4-mediated activation of NF-κB and release of TNF-α and IL-1β.

## Inductive effect of TLR4 activation on miR-155

On the other hand, miRNAs are also modifiable molecular targets. Accumulative evidences [91, 93, 100, 103–131] have indicated the role of miR-155 as one of TLR4-responsive miRNAs. It has long been recognized that miR-155 was processed from B-cell integration cluster (BIC) transcripts (or pri-155) and was highly induced in immune cells in response to various TLR ligands [103, 104]. In particular, the inductive effect of specific TLR4 ligand LPS on miR-155 has been verified in various in-vitro experiments using primary cells and immortalized cell lines (Table 1). In mouse Raw264.7 macrophages, BMDMs and human PBMCs, the in-vitro stimulation with LPS caused significant increases in the transcript expression levels of miR-155, which was on the top of the list of LPS-induced microRNAs identified by miRNA microarrays [93]. These increases were further validated by Northern blot and qPCR analyses [93, 105, 106]. In addition, utilizing the same analysis methods, the increased expression of miR-155 was also observed in human/murine primary DCs activated with LPS [107, 108]. Using a semi-quantitative RT-PCR method, Lu et al. [109] showed that the expression levels of both mature and precursor forms of miR-155 were robustly induced in LPS-treated primary human monocytes. In Raw264.7 cells, luciferase assay showed that LPS could drive the induction of pri-155 promoter in a dose-dependent manner, indicating that miR-155 might be regulated by LPS at the transcriptional level [110]. Treatment of mouse microglia N9 cells with increasing concentrations of LPS resulted in a significant and dose-dependent increase of miR-155 expression, which reached a 25-fold increase at the highest LPS concentration tested [111]. Similarly, a significant increase of miR-155 expression was also found in LPS-stimulated mouse primary microglia or immortalized BV2 cells [112, 113]. In the KCs isolated from alcohol-fed and pair-fed mice, Bala et al. [100] found that the alcohol-induced expression of miR-155 was significantly amplified by in-vitro LPS stimulation when compared with pair-fed mice. Besides, LPS-induced upregulation of miR-155 has also been demonstrated in non-immune cells. Treatment of human proximal tubule epithelial HK-2 cells with LPS for 24 h resulted in a significantly elevated expression of miR-155 [114, 115]. LPS increased expression levels of miR-155 in rat primary synovial fibroblasts, an in-vitro model for rheumatoid arthritis [116]. Treating human primary umbilical vein endothelial cells with LPS (0.05–1 μg/L) for 24 h enhanced the expression of miR-155 in a dose-dependent manner [117]. In mouse primary osteoblasts and osteoclasts, miR-155 expression was found to be upregulated in a time-dependent manner upon LPS stimulation [118, 119]. The similar upregulation of miR-155 upon LPS stimulation was also found in several tumor cell lines, including human breast cancer MCF-7 cells, B-lymphoma BJAB cells and mouse insulinoma MIN6 cells [120–122]. In order to clarify whether LPS-induced miR-155 expression is TLR4-dependent, De Santis et al. [123] treated BMDMs derived from WT-and TLR4^−/−^ mice with LPS for 2 h, and showed that the expression of miR-155 was upregulated in WT-BMDMs by 40-fold, but was decreased to less than twofold in TLR4^−/−^-BMDMs. Liu et al. [124] treated human bronchial epithelial 16HBE cells with TLR4 inhibitor prior to exposure to LPS, and found that LPS-induced upregulation of miR-155 was significantly attenuated by TLR4 inhibition.

In addition, the inductive effect of LPS on miR-155 has also been frequently verified in vivo (Table 1). Using the LPS-induced sepsis model, several studies [113, 125–127] have observed the significant increases of miR-155 expression in the brain, myocardium, liver and kidney tissues of mice challenged with LPS. Fu et al. [91] reported that LPS further enhanced the upregulation of miR-155 in the prostatic tissues of mice with experimental autoimmune prostatitis. In a LPS-induced acute lung injury animal model, miR-155 expression was found to be significantly upregulated in the lungs of mice and rats, and reached a peak at 6 h of LPS stimulation [128, 129]. Bala et al. [130] also observed an increase of miR-155 levels in the livers of WT mice following LPS or alcohol treatment, which was significantly eliminated by knockout of TLR4.

Despite the fact that there are lack of experiments on the human intestinal cells, e.g. Caco-2, which is originally derived from a colon carcinoma, the above evidences on the link between miR-155 and TLR4 from studies on human epithelial cell lines, including human proximal tubule epithelial HK-2 cells [114, 115], human breast cancer MCF-7 cells [120] and human bronchial epithelial 16HBE cells [124], laid the foundation for the possible existence of this interaction in the intestinal mucosa. Notably, Pathak et al. [131] reported that LPS increased miR-155 levels in intestinal fibroblasts and myofibroblasts isolated from health colonic mucosa, and this increase was further enhanced in those from UC patients, which suggested that the above link between miR-155 and TLR4 existed in the context of colitis and might play a role in colitis development.

Based on the above findings on the inductive effect of TLR4 activation on miR-155 expression, several studies [132–136] have been conducted to explore the possible underlying molecular mechanisms. Nuclear factor-κB (NF-κB) and activator protein 1 (AP-1) have been identified as important transcription factors downstream of TLR4 [32]. Arboleda et al. [132] showed that inhibition of NF-κB by SC-514, an inhibitor of IKK-β (a subunit of the NF-κB activation complex), diminished LPS-induced increase of miR-155-5p levels in human monocyte-derived macrophages. Using different NF-κB inhibitors, this finding was validated in LPS-treated Raw264.7 cells and mouse KCs [133, 134]. Dai et al. [134] demonstrated that NF-κB p65 and AP-1 family members JunB, FosB directly bound to the BIC/miR-155 promoter and promoted the transcription of miR-155, which were detected using DNA precipitation assay and luciferase assay, respectively, in human trophoblast cell HTR-8/SVneo after LPS treatment. In Raw264.7 cells, McCoy et al. [135] found that the transcription factor AP-1 might be required for BIC gene induction in response to LPS stimulation, thus play a role in the LPS induction of miR-155. In addition, Quinn et al. [110] reported that miR-155 promoter was controlled by Ets family of transcription factors, in which Ets2 was strongly induced by LPS. This study team also found that LPS-induced pri-miR-155 expression was significantly reduced in macrophages isolated from the Ets2^−/−^ mice, verifying the importance of Ets2 in LPS-mediated induction of miR-155. Moreover, knockdown of KH-type splicing regulatory protein (KSRP, a single‐strand RNA‐binding protein that bound to the terminal loop of miRNA precursors and promoted their maturation) could cause a decrease of mature miR-155 expression in LPS-treated BMDMs and Raw264.7 macrophages, whereas the expression of pri-miR-155 was not affected, suggesting that post-transcriptional mechanisms might also be involved in LPS-induced miR-155 expression [136].

## A possible TLR4-miR-155 feedback loop in CAC

**Fig. 1:**
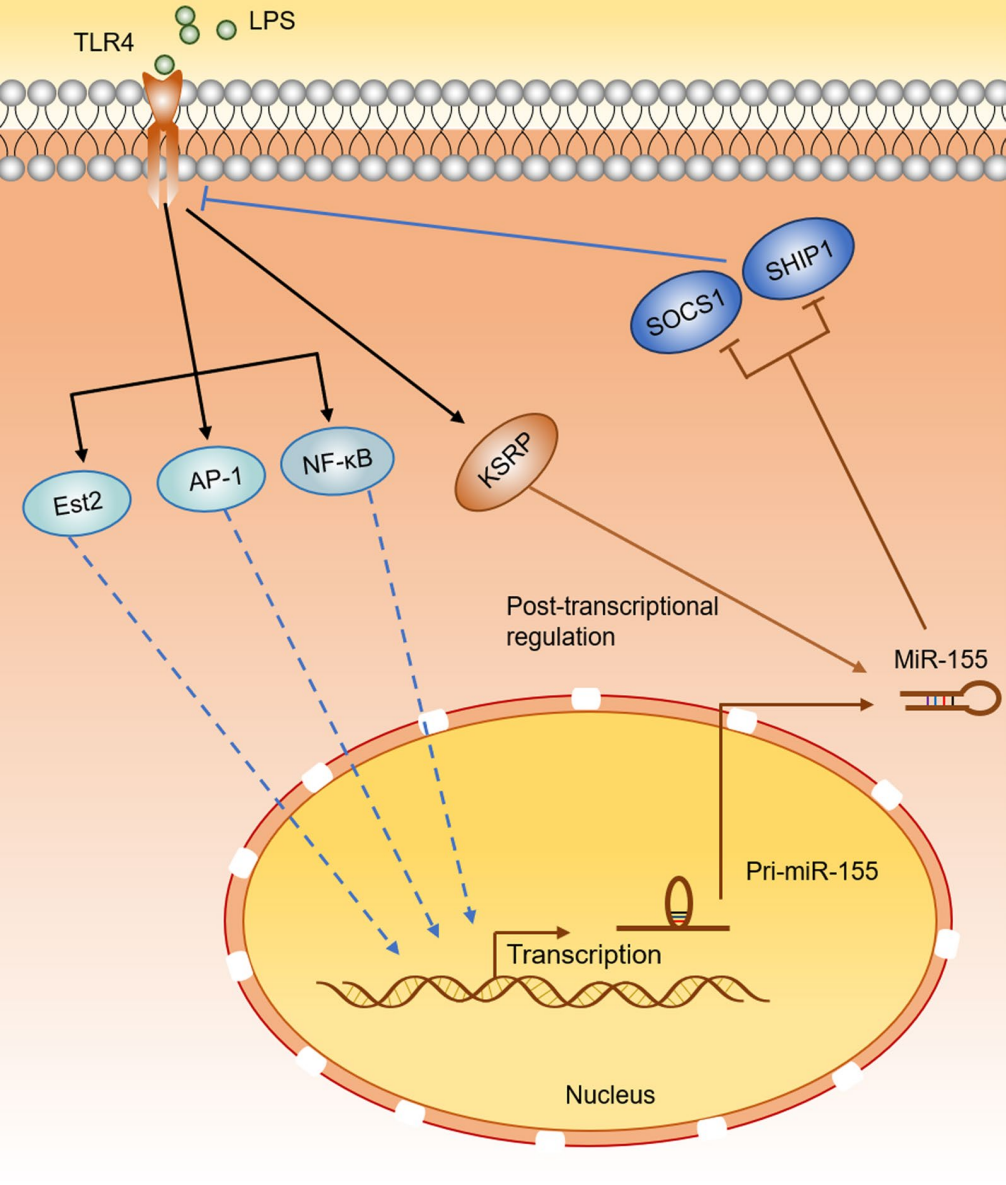
Model of possible TLR4-miR-155 positive feedback loop. LPS-activated TLR4 signaling might promote the transcription of pri-miR-155 by triggering transcription factors such as NF-kB, AP-1, Ets2, and might enhance the maturation of miR-155 through a post-transcriptional mechanism depending on the induction of KSRP. These events might result in the overexpression of miR-155. The high level of expressed miR-155 might target SOCS1 and SHIP1, two key negative regulators of TLR4 signaling, thereby promoting the expression of TLR4, and ultimately constituted a TLR4-miR-155 positive feedback loop

Collectively, in the context of different disorders or cell lines, miR-155 has been extensively found to act as a potential TLR4 signaling upregulation mechanism. While TLR4 activation would instead enhance the miR-155 expression to constitute a TLR4-miR-155 positive feedback loop (Fig. 1). Once this feedback loop could be verified in the context of CAC, based on the similar dynamic expression changes of miR-155 and TLR4 during CAC development, as well as their similar functions in CAC promotion, it could be speculated that this TLR4-miR-155 positive feedback loop would lead to the maintenance and amplification of oncogenic effects of TLR4 signaling, ultimately resulting in the synergistic accelerating effect of TLR4 and miR-155 on CAC development.

## Limitations and further directions

About 20 miRNAs have identified to be involved in the regulation of TLR signaling pathways [137]. Among them, miR-155, miR-21 and miR-146a are the three miRNAs that received extensive attention due to their sensitive expression changes following TLRs activation and their regulatory roles in TLRs signaling [88]. Especially, miR-155 and miR-21 are also considered as the most prominent miRNAs playing central roles in molecular dysfunctions linking inflammation with cancer [138]. Based on the more abundant evidences on potential link between TLR4 and miR-155, as well as their similar dynamic expression changes during CAC development and their similar CAC-promoting effects, this review focused only on miR-155, in an attempt to present a reasonable hypothesis for the potential association between TLR4 and miR-155 in the context of CAC. Nevertheless, other TLR4-related miRNAs and their potential roles in CAC should be further summarized. For example, the negative regulation of TLR4 via targeting of the proinflammatory tumor suppressor PDCD4 by the miR-21 in CRC has been reported [138]. However, other studies [139, 140] reported that miR-21 acted as one of the positive factors that trigger the inflammatory feedback loop, thus induced and maintained the transformed state. Accordingly, miR-21 has been believed to be involved in both positive and negative feedback loops that control inflammation and CRC [138].

## Conclusions

In summary, both clinical and experimental evidences showed a gradual increase of TLR4 expression during different stages of CAC development. As a classic PRR, TLR4 might act as a key bridge molecule between the infection of oncogenic pathogens (such as *F. nucleatum* and *Salmonella)* and the colonic inflammatory process. More importantly, the upregulated TLR4 in colitis could play an important role on inflammation-induced intestinal tumorigenesis by promoting cell proliferation, protecting malignant cells against apoptosis, facilitating invasion and metastasis, as well as contributing to the creation of tumor-favouring cellular microenvironment. In particular, this review highlights the interplay between TLR4 and miR-155, a CAC-related miRNA. These two molecules shared highly similar dynamic changes during stages of CAC development as well as highly similar CAC-promoting effects. In the context of different disorders or cell lines, miR-155 has been found to augment TLR4 signaling through targeting negative regulators SOCS1 and SHIP1; and TLR4 activation could induce miR-155 expression via transcriptional and post-transcriptional mechanisms, which might constitute a TLR4-miR-155 positive feedback loop. The further studies on this TLR4/miR-155 interplay in the context of CAC would facilitate the development of novel strategies for CAC prevention and control [67].

## Data Availability

This article reviews literature and therefore does not contain any associated data and materials.

## Abbreviations

AOM: Azoxymethane
AP-1: Activator protein 1
Bcl-2: B-cell lymphoma 2
BIC: B-cell integration cluster
BM: Bone marrow
BMDMs: Bone marrow derived macrophages
CAC: Colitis-associated cancer
CCL: C–C motif chemokine ligand
CD: Crohn’s disease
CD14: Cluster of differentiation 14
Cox-2: Cyclooxygenase 2
CRC: Colorectal cancer
CX3CL1: Chemokine C-X3-C-Motif Ligand 1
DCs: Dendritic cells
DSS: Dextran sulfate sodium
EMT: Epithelial-mesenchymal transition
FU: Fluorouracil
IBD: Inflammatory bowel diseases
IL: Interleukin
KCs: Kupffer cells
KSRP: KH-type splicing regulatory protein
LBP: LPS binding protein
IMF: Intestinal myofibroblasts
LPS: Lipopolysaccharide
MD-2: Myeloid differentiation factor 2
miR-155: MicroRNA-155
MyD88: Myeloid differentiation primary response gene 88
NF-κB: Nuclear factor-κB
NO: Nitric oxide
OGD: Oxygen–glucose deprivation
PAMPs: Pathogen-associated molecular patterns
PPRs: Pattern-recognition receptors
PSC: Primary sclerosing cholangitis
SHIP1: Src homology 2 domain-containing inositol-5′-phosphatase 1
SOCS1: Suppressor of cytokine signaling 1
STAT: Signal transducer and activator of transcription
TLR4: Toll like receptor 4
TNF-α: Tumor necrosis factor-α
TRAIL: TNF-related apoptosis-inducing ligand
TRAM: TRIF-related adaptor molecule
TRIF: TIR-domain-containing adapter-inducing interferon-β
UC: Ulcerative colitis
UTRs: 3′-Untranslated regions
WT: Wild type
